# Dietary Intake of Vitamin D in the Czech Population: A Comparison with Dietary Reference Values, Main Food Sources Identified by a Total Diet Study

**DOI:** 10.3390/nu10101452

**Published:** 2018-10-07

**Authors:** Svatava Bischofova, Marcela Dofkova, Jitka Blahova, Radek Kavrik, Jana Nevrla, Irena Rehurkova, Jiri Ruprich

**Affiliations:** 1 Centre for Health, Nutrition and Food, NIPH—National Institute of Public Health in Prague, 612 42 Brno, Czech Republic; dofkova@chpr.szu.cz (M.D.); blahova@chpr.szu.cz (J.B.); kavrik@chpr.szu.cz (R.K.); nevrla@chpr.szu.cz (J.N.); rehurkova@chpr.szu.cz (I.R.); jruprich@chpr.szu.cz (J.R.); 2 Department of Public Health, Faculty of Medicine, Masaryk University, 625 00 Brno, Czech Republic; 3 Department of Milk Hygiene and Technology, Faculty of Veterinary Hygiene and Ecology, University of Veterinary and Pharmaceutical Sciences, 612 42 Brno, Czech Republic

**Keywords:** vitamin D, dietary intake, Czech population, micronutrient adequacy, dietary sources, total diet study

## Abstract

The usual dietary intake of vitamin D was studied in 10 subgroups of the Czech population. Food consumption data was collected using repeated 24 h recall in a national cross-sectional survey (the Study of Individual Food Consumption, SISP04), and the vitamin D content in marketed foods was quantified within the national Total Diet Study (2014–2015). The Monte Carlo Risk Assessment computational model (version MCRA 8.2) was used to assess usual intake. The median vitamin D intakes for the Czech population (aged 4–90 years, both genders) were within a range of 2.5–5.1 μg/day. The highest median intake, excluding dietary supplements, was observed in men aged 18–64, and the lowest was observed in children aged 4–6 and girls aged 11–17. The main sources in the diet were hen eggs (21–28% of usual dietary intake), fine bakery wares (11–19%), cow’s milk and dairy products (7–23%), meat and meat products (4–12%), fish (6–20%), and margarines (7–18%). The dietary intake of vitamin D for more than 95% of the Czech population was below the recommended Dietary Reference Values (DRVs). These findings should encourage public health authorities to support interventions and education and implement new regulatory measures for improving intake.

## 1. Introduction

Vitamin D deficiency is recognized as a global health problem at the level of all population subgroups [1]. It is associated not only with weak musculoskeletal health (e.g., rickets in children, osteopenia, osteoporosis and fractures in adults etc.), but also with increased risk of other illnesses (e.g., common cancers, autoimmune diseases, hypertension, infectious diseases, and some neuropsychological diseases) [2]. Vitamin D receptors are situated in almost every cell and tissue, evidencing its important role in many body systems (the immune, cardiovascular, and nervous systems etc.). There are two main forms of vitamin D: vitamin D_3_ (referred as cholecalciferol) and vitamin D_2_ (referred as ergocalciferol). For human beings, vitamin D_3_ is synthesized endogenously in the skin during ultraviolet B (UVB) exposure or through diet, whereas vitamin D_2_ is delivered only through the diet.

Endogenous synthesis may be the main source of vitamin D, but it is influenced by many factors (latitude, season, time of day, ozone layer, air pollution, clouds, surface, time spent outdoors, use of sunscreen, clothing, skin color, age, overweight/obese status, health conditions, and others) [3]. Vitamin D synthesis ceases between October and March in the Northern Hemisphere at latitudes greater than 40° north. Since the Czech Republic is situated at a latitude of around 50° north, dietary intake becomes an essential source, at least during the winter season [1].

Vitamin D is naturally present in a limited number of foods, but fortified foods or dietary supplements could be also good dietary sources. The high content of vitamin D is typical for fatty fish (0.7–19 μg/100 g), because fish accumulates provitamins of vitamin D, vitamin D_2_ and D_3_, from phytoplankton and zooplankton. Egg yolks are also good dietary source. The vitamin D content in egg yolk depends mostly on the composition of hen feed and reaches up to 12.6 μg/100 g [4]. Other animal sources of vitamin D_3_ are liver, meat and meat products, and milk and dairy products. Mushrooms can also be a source of vitamin D. Vitamin D_2_ is produced on UVB exposure of the precursor vitamin D_2_ (ergosterol). Vitamin D_2_ content ranges from 1 to 30 μg per 100 g fresh weight in wild mushrooms [5]. Cultivated mushrooms produced indoors do not contain vitamin D without UVB irradiation. New pure yeast or bread (containing ergosterol) treated with UVB light may be also a source.

Due to the pleiotropic effects of vitamin D in organisms as investigated over the last 20 years, Dietary Reference Values (DRVs) for dietary intake have been revaluated recently. The United States Institute of Medicine (IoM US) updated DRVs for vitamin D in 2011. The Estimated Average Requirement (EAR) was set for individuals aged one year and over at 10 μg/day and the Recommended Dietary Allowance (RDA) was set at 15 μg/day for those aged 1–70 years and 20 μg/day for those aged 71 years and older, based on conditions of minimal sun exposure [6]. Even these increased DRVs are considered too low [7]. The European Food Safety Authority (EFSA, 2016) set an Adequate Intake (AI) of vitamin D at 15 μg/day for individuals aged one year and older under conditions of assumed minimal cutaneous vitamin D synthesis [3]. The German-speaking countries (DACH 2015: Deutschland, Austria, Confoederatio Helvetica) neighboring the Czech Republic set the AI of vitamin D for people aged one year and over at 20 μg/day in the case of lack of endogenous synthesis [8].

Since there has been an absence of new up-to-date data on vitamin D content in foods marketed in the Czech Republic, relevant intakes and dietary sources in different population groups remain unknown. However, this information is necessary for understanding the situation in the country and for proper decision-making by public health authorities. Thus, it was decided to include vitamin D into the ongoing national Total Diet Study (TDS) because it is widely recognized as an effective and adequately efficient tool to gain reliable data on the occurrence of chemical substances across diet and to estimate dietary exposure or intake in a population [9]. The aim of this article is therefore to describe the usual dietary intake of vitamin D in 10 Czech population groups (aged 4–90 years) based on currently measured vitamin D content in marketed foods within the national TDS, in order to compare results with available DRVs and to define the main exposure sources of vitamin D in the habitual diet of the Czech population.

## 2. Materials and Methods

The TDS method combines individual food consumption data with directly measured concentrations of vitamin D in pooled staple foods representing habitual diets. The TDS was followed by modeling of exposure doses to estimate dietary intake and identification of staple food contributors.

### 2.1. Data on Food Consumption

The food consumption data used for evaluation of vitamin D intake originated from the most recent national cross-sectional dietary survey (the Study of Individual Food Consumption, SISP04) performed in the Czech Republic over 2003–2004. The SISP04 was carried out using repeated 24 h diet recall with an age- and gender-representative sample of the Czech population. The used methodology respected recommendations for conducting a national dietary survey [10]. The sample was selected by multistage sampling combining random and quota selection. The National Population and Housing Census in 2001 served as the sampling frame. Institutionalized, homeless people and people speaking languages other than Czech or Slovak (<4%) were not included. Two nonconsecutive 24 h recalls were obtained from each participant through face-to-face interviews conducted by trained interviewers in participants’ households. Children below the age of 15 were interviewed in the presence of parents and small children with parental assistance. The time span between both interviews was from one to six months. Data on consumption were collected during the whole year to reduce the influence of seasonality in food consumption. All days of the week were covered by the sampling calendar. To quantify amounts of consumed food, a picture book and household measures were used. Meals were decomposed into ingredients according to individual or standard recipes. The SISP04 includes the data on food consumption from 2590 individuals (males and females aged 4–90 years, see Table 1). The overall response rate was 54%. To check the quality of collected data the Goldberg/Black equation was applied for evaluation of reported energy intakes at the individual level [11]. More details on SISP04 methodology and specific data on food consumption can be found elsewhere [12,13,14]. Data on consumption from two nonconsecutive days were used for estimation of usual (habitual) vitamin D distribution in population subgroups. Two nonconsecutive days are sufficient for this purpose when combined with statistical modeling [15,16].

### 2.2. Data on Vitamin D Content in Foods

The vitamin D content in foods was determined by analytical measurement as a part of the national TDS. The TDS has been conducted in the Czech Republic since 1994 and covers not only contaminants but also beneficial substances in the diet. Its methodology respects the international guidelines for implementation of a TDS [9]. Vitamin D assessment was included in the national TDS in the two-year cycle from 2014 to 2015. The TDS considers the consumer behavior model which means that sampled foods include a wide range of foods usually consumed by a population and foods are culinary treated (processing the food as for consumption) before chemical analysis. Sampling in the national TDS was based on results of the national dietary survey (SISP04) and covers more than 95% staple foods of the Czech diet. The collection of food samples for vitamin D analysis was carried out in retail shops according to a complex sampling plan in 32 different locations of the Czech Republic. The selection of shops and time of purchase (seasons) respected the consumer behavior of the Czech population regularly described in household budget surveys. In total, 132 different foods (in 12 subsamples) which represented possible sources of vitamin D in the diet according to the food compositional tables were sampled. Foods were registered, culinary treated according to standardized recipes reflecting the preferred kitchen preparation by the Czech population, and pooled into 86 pooled samples before analysis. Together, 356 pooled samples composed of 4346 individual food subsamples were analyzed, with at least four for each. To ensure the accuracy and precision of food sampling and preanalytical preparation of samples, all steps were described and documented in standard operating procedures. The detailed methodology of food sampling in the national TDS is given elsewhere [17,18]. The usual intake of vitamin D does not include intake from dietary supplements, only that from staple foods.

### 2.3. Analytical Measurement of Vitamin D in Foods

Liquid chromatography-mass spectrometry (LC-MS/MS) was used for the determination of seven chemical individuals of vitamin D and its metabolites (ergocalciferol (vitamin D_2_); 25-hydroxyergocalciferol; 1,25-dihydroxyergocalciferol; cholecalciferol (vitamin D_3)_; 1,25 dihydroxycholecalciferol; 24,25-dihydroxycholecalciferol; and 25-hydroxycholecalciferol) in foods. The measurements were carried out in the accredited laboratory (according to the European Standard EN ISO/IEC 17 025:2005, general requirements for the competence of testing and calibration laboratories) of the Centre for Health, Nutrition and Food (National Institute of Public Health).

Culinary treated TDS samples were homogenized and stored at −80 °C in a deep-freezing box (for no more than three months) and defrosted at laboratory temperature before the analysis. Sample preparation was based on alkaline saponification and then extraction of the analytes into non-polar solvent (hexane). The weight of the analytical portion ranged between 0.1 and 1.0 g (depending on the fat content and the presumed vitamin D content). Each sample was analyzed in a triplicate.

An Agilent 1200 series liquid chromatograph (Agilent Technologies, Santa Clara, CA, USA) in tandem with a triple-quadrupole API 4000 mass spectrometer (Applied Biosystems, Foster City, CA, USA) was used for the determination of vitamin D and its metabolites in foods. Positive ion Atmospheric Pressure Chemical Ionization (APCI) was used as an ionization technique. A Kinetex PFP chromatographic column 50 × 2.1 mm with a particle size of 2.6 μm was used to separate the analytes. Here, 0.1% HCOOH in water (solvent A) and 0.1% HCOOH in MeOH (solvent B) were used as mobile phase in gradient mode. Established Multiple Reaction Monitoring (MRM) transitions of analytes of interest were as follows: vitamin D_2_—397/379; vitamin D_3_—385/259; 25-OH D_2_—413/355; 25-OH D_3_—401/365; 1,25-OH D_2_—429/411; 1,25-OH D_3—_417/399; and 24,25-OH D_3_—417/381 [19].

The analytical method was optimized and validated. Limits of quantification (LOQ; μg/kg) were determined as follows: vitamin D_2_—0.39; vitamin D_3_—0.18; 25-OH D_2_—0.84; 25-OH D_3_—0.97; 1,25-OH D_2_—0.69; 1,25-OH D_3_—0.86; and 24,25-OH D_3_—0.25. Recovery of all analytes was verified on a set of real samples representing different matrices, which were spiked by the mixture of standards of all analytes. Recovery ranged between 70 and 110% according to the matrix.

The accuracy and precision of the entire analytical procedure were controlled by using the composite internal standard containing isotopically-labeled (C13) vitamin D_3_, 25-OH D_3_, and 1,25-OH D_3_ by measuring the certified reference material SRM 1849a Infant Formula (NIST) in each series, and by successfully participating in proficiency testing (FAPAS, Food Analysis Performance Assessment Scheme, Fera Science Ltd., UK).

### 2.4. Estimation of Vitamin D Usual Dietary Intake in the Czech Population

Modeling of vitamin D dietary intake was based on individual food consumption data and TDS concentration data. The Monte Carlo Risk Assessment computational model (MCRA, version 8.2) [20] was used to estimate usual dietary intake. We applied following calculation parameters: MCRA settings for the TDS, the Logistic-Normal-Normal exposure model, and the lower bound (LB) and upper bound (UB) for intake of the sum of vitamin D_2_ + D_3_. Lower Bound left-censored concentration values for vitamin D_2_ or D_3_ were replaced by 0, and the UB were replaced by the LOQ for each analyzed food sample. The 5th, 25th, 50th, 75th, and 95th percentiles of intakes and their confidential intervals (CI p2.5 and p97.5) per person and day were calculated. Dietary intakes of vitamin D were estimated specifically for 10 Czech population groups: children (both sexes) aged 4–6 years, children (both sexes) aged 7–10 years, males aged 11–14 years, females aged 11–14 years, males aged 15–17 years, females aged 15–17 years, males aged 18–64 years, females aged 18–64 years, males aged ≥65 years, and females aged ≥65 years. The choice of these population groups was related to the structure of results from the national food consumption study and recommendations for vitamin D dietary intake.

### 2.5. Assessment of Vitamin D Dietary Intake

To evaluate the adequacy, the vitamin D usual intake distributions were compared with the American EAR (IoM US 2011) and European AI (EFSA 2016, DACH 2015) reference values. The EAR cut-point method was used when comparing vitamin D usual intake with the EAR. This approach assumes that proportion of individuals with usual intake below the EAR in a group corresponds to the proportion of subjects with inadequate intake. The comparison with AI reference values can be performed qualitatively. If the median intake is above the AI, the prevalence of inadequate intake in a population could be considered as low. However, if the median intake is below the AI, the adequacy cannot not be evaluated [6,21].

### 2.6. Limitation of Study Results

Among the limitations of the results it should be mentioned that a relatively long time had passed since the data on food consumption (SISP04) was collected. However, it is necessary to highlight that it was the most recent national-wide survey on the individual level conducted in the Czech Republic. Food consumption patterns over time seemed quite unchanged in the country based on Czech Statistical Office data comparing the years from 2006 to 2014 [22]. Moreover, the sampling in the national TDS is regularly updated based on market share data to avoid this disadvantage. Another possible source of inaccuracy is the fact that foods sampled in the national TDS represent not the whole usual Czech diet but only about 95%.

## 3. Results

### 3.1. Usual Dietary Intake and Its Distribution in Ten Czech Population Groups

Usual vitamin D intakes from foods (without dietary supplements) were low in the Czech population. The median usual intakes ranged from 2.5 μg/day (in children 4–6 years, in females 11–14 years, and in females 15–17 years) to 5.1 μg/day (in men aged 18–64 years). When compared to the EFSA and DACH DRV, the adequacy of vitamin D intake could not be evaluated, because the values of mean intake were below the AI. However, the vitamin D intake was evaluated as inadequate in all population subgroups when compared with EARs of the Institute of Medicine [6]. The deficiency of vitamin D in the diet was observed in more than 95% of individuals in all evaluated population groups.

The distribution of usual dietary intakes found in TDS for the 10 Czech population groups and the comparison with DRVs (AI, EAR) are presented in Table 2.

### 3.2. Food Sources of Vitamin D Intake in the Czech Population Groups

Table 3 shows main exposure dietary sources of vitamin D in the Czech Republic as recognized by the TDS. In all population groups hen eggs, fine bakery wares (excluding biscuits), and margarines were identified among the most important sources of dietary vitamin D.

### 3.3. Vitamin D Content in Foods and Food Groups

In total, 86 pooled types of food samples were analyzed for vitamin D content. Only two forms of vitamin D (D_2_ and D_3_) were detected in foods; other forms were always below the limits of detection. Vitamin D_2_ was detected only in low concentrations in a minority of TDS samples (e.g., in marinated fish, smoked fish, and canned meat) and therefore it probably does not play too important a role in the dietary intake of Czechs.

**Figure 1:**
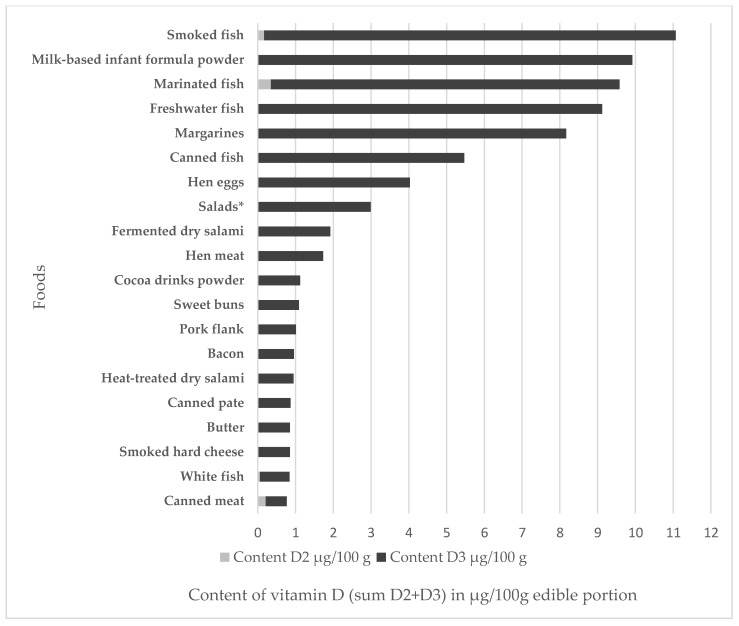
The 20 foods with the highest vitamin D content. * This pooled sample is for 50% fish salad.

Figure 1 shows 20 groups of foods (TDS pooled samples) with the highest average content of vitamin D (sum of D_2_ + D_3_) in 100 g of edible portions. The highest contents of vitamin D (sum of D_2_ + D_3_) were measured in fish and fish products (smoked, marinated, freshwater, canned fish) at 5.45–11.11 μg/100 g, in milk-based infant formula powder at 9.92 μg/100 g, in margarines (which are fortified foods) at 8.17 μg/100, in hen eggs at 4.03 μg/100 g, in salads (composed of 50% fish salad) at 2.99 μg/100 g, in fermented dry salami at 1.93 μg/100 g, and in hen meat at 1.73 μg/100 g.

Table 4 shows an overview of analyzed food groups and the ranges of vitamin D content. In total 86 pooled samples, which included 132 food items, were divided into 19 groups. This division is based on the recommended FoodEx2 classification [23].

## 4. Discussion

Vitamin D in the body originates either from dietary sources or from endogenous synthesis. As mentioned previously, due to its geographical location the Czech population is to a large extent dependent on dietary intake of vitamin D at least during the late autumn, winter, and early spring. Moreover, some population groups can be at risk year-round (i.e., people with minimal sun exposure—e.g., individuals working indoors, homebound individuals, institutionalized people, etc.). That is why the present research was focused on describing vitamin D intake from diet. The results describe usual dietary intake of vitamin D in the Czech population, which was evaluated on the basis of individual food consumption data from the most recent national dietary survey (SISP04) and measured concentrations of vitamin D in marketed food sampled as a part of the national TDS. Such approach was more advantageous compared to the usage of Food Composition Tables (FCTs) for calculation of dietary intake because data on vitamin D content in Czech FCTs is not entirely up to date or is missing for some foods. Intake from dietary supplements is not included into presented results because only limited information on that was available in food consumption data.

It was found that median usual intakes of vitamin D from foods ranged from 2.5 to 4.9 μg/day in Czech children and adolescents (aged 4–17 years) and from 2.8 to 5.1 μg/day in Czech adults (aged 18–90 years). The highest dietary intake of vitamin D (9.9 μg/day, UB, 95th percentile) was observed in men aged 18–64 years.

When comparing the Czech results with those from other European countries the situation in terms of vitamin D dietary intake is similar. According to an EFSA report (2012) comparing results from 14 European countries, the mean intake without dietary supplements in children and adolescents varied from 1.4 to 4 μg/day, in adult women from 1.1 to 6.0 μg/day, and in adult men from 1.5 to 8.2 μg/day. The highest intake from foods was identified in men from Finland (16 μg/day, 25–74 years, 95th percentile) [24]. In this context it is necessary to mention that there was a large diversity in the methodologies of the described studies.

Vitamin D intake, excluding food supplements, was also explored in the EPIC (the European Prospective Investigation into Cancer and Nutrition) study (1992–1999) in 10 European countries using 24 h dietary recall and a standardized nutrient database. When all countries were combined, the mean vitamin D intake (the EPIC mean) was determined in adults (35–74 years) as 3.3 μg/day for women and 4.8 μg/day for men, and the highest level of vitamin D intake was observed in Sweden [25].

Viñas (2011) compared vitamin D intake in European adults (19–64 years) and the elderly (>64 years) from 11 European countries. Mean daily vitamin D intakes were between 2 and 5 μg/day in most investigated countries, but the highest (up to 9 μg/day) was in Nordic countries (Sweden, Finland) [1,26]. Mensink et al. (2013) reanalyzed raw data from eight European national surveys (Belgium, Denmark, France, Germany, the Netherlands, Poland, Spain, and the United Kingdom). The mean intakes ranged in children and adolescents (4–17 years) from 1.5 to 4.8 μg/day, and in adults from 0.8 to 6 μg/day [27]. The most recent data from a Spanish study (ANIBES) showed the total mean intake of vitamin D in the population was 4.4 ± 0.1 μg/day [28], and a similar Belgian study (VITADEK) showed the mean intake of vitamin D from natural and fortified foods (without supplements) was 3.37–4.41 μg/day in men aged 3–64 years and 3.18–3.48 μg/day in women aged 3–64 years [29].

In most of the above referenced surveys the highest levels of vitamin D intake were described in Nordic countries. The difference between Nordic countries and the Czech Republic can be attributed especially to much lower fish consumption among Czech people. Moreover, a much wider range of products in Nordic countries is mandatorily fortified (milk, milk products, margarine) [1] when compared to the situation in the Czech market.

A comparison with available DRVs indicated that the dietary intake of vitamin D is too low in the majority of the Czech population (>95%). Unfortunately, there was only a limited possibility for comparison of the present findings with results on vitamin D status based on biomarker measurements such as 25-hydroxyvitamin D (25(OH)D). The main marker of vitamin D saturation in the body is 25(OH)D, which reflects dietary intake as well as endogenous synthesis. Some data were produced as a part of the Czech biological monitoring performed by the NIPH, but its status has only been described recently in children aged 5 and 9 years (a study for adults is ongoing). In the spring about 50% of children (*n* = 124) had 25(OH)D levels lower than 50 nmol/L; in summer this was the case for about 10% of children (*n* = 85), and in winter for more than 30% of children (*n* = 66) [30]. Even those these results are not extensive, they support our findings that dietary intake is low and, particularly in the winter season, it is not enough to cover vitamin D requirements.

The foods that contribute substantially to dietary intakes of vitamin D differ across countries according to habitual dietary patterns and fortification policies. In the Czech population the following main sources of vitamin D exposure were identified: hen eggs (contribution to the usual intake: 21–28%), fine bakery wares excluding biscuits (11–19%), cow’s milk and dairy products (7–23%), fortified margarines (7–18%), fish and fish products (6–20%), and meat and meat products (4–12%).

In the United Kingdom, the major dietary sources in children aged 4–10 years are milk and milk products (13%), meat and meat dishes (25%), fat spreads (21%), cereal and cereal products (20%), fish and eggs (8%). In adults the main dietary sources of vitamin D are meat and meat products (27% for women, 34% for men), fat spreads (19% for men, 20% for women), fish and fish dishes (15% for men, 18% for women), eggs (14% for men, 12% for women), cereal and cereal products (13% for men, 12% for women), and milk and milk products (5% in both groups) [31]. In Irish adults, the significant contributors are meat (30% in people aged 18–64 years, 22% in people ≥65 years), fish (12% in people aged 18–64 years, 16% in people ≥65 years), and spreads (10% in people aged 18–64 years, 13 % in people ≥65 years) [32]. In Denmark, in the population aged 7–39 years, the main contributors of vitamin D are fats (36%), meat and meat products (20%), fish and shellfish (8%), and fine pastries (7%) [21]. In France, fish contributes 31% of dietary vitamin D intake in children and 38% in adults. Eggs contribute 10% and 9%, and cheese 9% and 7% of dietary vitamin D intake in children and adults, respectively [33]. In Spain, the situation is similar to that of France. The main source of vitamin D is fish (68% of vitamin D intake), eggs (20%), and cereals (4%) [34]. In Finland, the major food sources of vitamin D are fish, dietary fats, and fortified liquid milk and dairy products. Dietary fats have been fortified with vitamin D since the 1950s and milk since 2003 [35,36]. In Norway, the most important sources of vitamin D are fatty fish, fortified fats (margarines, butter), and supplements with cod liver oil, a similar situation to that in Iceland. In Sweden, the main vitamin D sources are dietary fat, oil-rich fish and fish products, and fortified milk and dairy products [1,37].

As we can see, fatty fish are an important source of vitamin D and they contribute up to 68% of the total dietary intake in some countries. However, in the Czech Republic fatty fish contribute 20% at maximum (in the group of women aged 18–64 years). Although the Czech Nutrition Society (2012) recommends a weekly intake at least 400 g of fish and fish products [38], fish consumption is still low in the long term. Fish consumption was only 5.5 kg (raw) per capita per year in 2015 [39] i.e., about 100 g weekly. This is probably due to the Czech Republic’s historical development, cultural traditions, and inland location. There are many attempts to increase fish consumption in the Czech population, for example, recommendations from professional authorities or media campaigns, but changing consumer habits is a long-term process. A wide range of fish and fish products is available on the Czech market, but it seems the specific fish characteristics (smell, relatively large numbers of small bones, higher price especially in sea fish etc.) could have a significant impact on the previously mentioned low consumption, mainly among children. Despite the described features, an increase in fish consumption in the Czech population is publicly supported because it is favorable not only for vitamin D but also for intake of other positive substances like omega 3 fatty acids. It should be mentioned that public health interventions to increase intake of vitamin D are not new. Children regularly received fish oil in schools during 1950s and early 1960s as a form of prevention of rickets.

Hen eggs, which are the major contributor to total vitamin D intake in the Czech Republic (up to 28%), accounted for 20% at maximum in other compared countries. The reasons for the higher contribution in the Czech Republic are the flat fortification of feed for laying hens and a relatively high egg consumption rate.

There are several possibilities for how to improve vitamin D intake in a population. One of the most commonly used solutions is fortification of foods. For example, fortified fats are an important source of vitamin D in many countries (Denmark, Nordic countries, and the Czech Republic). However, the policy of vitamin D fortification is not harmonized across Europe. In the Czech Republic, mandatory food fortification applies only for nutrition for infants and young children. Other foods (margarines, milk, instant cocoa drinks etc.) are fortified voluntarily. At present, there are not a lot of vitamin D fortified products on the Czech market; those are mostly produced by global food companies. The introduction of mandatory fortification of certain products or voluntary fortification of a larger range of products (e.g., milk, dairy products, breakfast cereals, pasta etc.) could contribute substantially to higher vitamin D intake on a population-wide basis in the Czech Republic. However, the remaining issue is affordability of such products. Currently, vitamin D fortified milk is often twice as expensive as non-fortified milk, and therefore not cost-attractive for consumers.

Another possibility how to increase dietary intake is introduction of foods (mushrooms, yeast, pastry with yeast e.g., bread), which naturally contain the vitamin D precursor (ergosterol) and are treated with UVB light. Therefore, food producers could be encouraged to use UVB in food production. Since this measure is a relatively new method for the increasing vitamin D content in foods, such products are referred to as novel foods and their placement on the market is subject to special procedure.

Dietary supplements can be also a means to improve vitamin D intake. There are differences in the usage rate of dietary supplements across Europe. Higher consumption is typical for Nordic countries compared to the south of Europe and there are differences also between genders. The consumption is higher in women than in men [1]. In the Czech Republic there is compulsory supplementation of vitamin D in infants and small children in order to prevent of rickets. In other population groups it is voluntary. According to limited information from national survey (SISP04) we can assume that the frequency of days of consumption of dietary supplements containing vitamin D is about 6% in adult men and about 11% in adult women.

## 5. Conclusions

The presented results indicate that in the Czech Republic there is a large percentage of people who do not meet the current recommended dietary intake for vitamin D. The prevalence of inadequate dietary intake is estimated to be at least 95% in the Czech population aged four and over. Small differences were observed in population groups, with the highest vitamin D intake being in adult men aged 18–64 years, and the lowest in children aged 4–6 years, girls aged 11–14 years, and women aged 15–17 years. Such findings should encourage public health authorities to support population interventions, consumer education, and possibly also implementation of regulatory measures for improving vitamin D intake, including not only voluntary food fortification.

## Figures and Tables

**Table 1:** Characteristics of studied Czech population groups.

Sex/Age Category	Number of Subjects	Characteristics of Age Groups According to Percentiles 25th–Mean–75th	Mean Body Weight (kg)	Mean Energy Intake (kcal·day^−1^) (Data Including Misreporters)
Children, 4–6 years	196	4.0–5.1–5.4	21.3	1903
Children, 7–10 years	311	7.4–8.8–9.2	32.6	2192
Males, 11–14 years	49	11.2–12.6–13.0	47.8	2891
Females, 11–14 years	51	11.0–12.4–13.0	46.6	2340
Males, 15–17 years	56	15.1–16.2–16.4	66.2	3474
Females, 15–17 years	58	15.0–16.1–16.4	55.8	2098
Males, 18–64 years	793	30.3–41.9–53.0	82.6	3148
Females, 18–64 years	873	31.5–44.0–55.5	69.1	2018
Males, ≥65 years	80	65.7–70.9–74.3	82.9	2660
Females, ≥65 years	123	66.8–71.8–75.3	73.3	1980

**Table 2:** Distribution of usual intake of vitamin D (sum of D_2_+D_3_ in μg/person/day) from total diet in 10 Czech population groups.

Sex/Age Category	*n*	Percentiles of Usual Intakes (LB-UB) ^(1)^					Requirement AI ^(2)^ (μg/day)		% Inadequacy ^(3)^	Requirement EAR	% of Individuals Exceeding the EAR (LB-UB Intakes) ^(4)^
		5	25	50	75	95					
		(μg/person/day)					EFSA * 2016	DACH * 2015		IoM US * 2011	
Children, both sexes, 4–6 years	196	1.6–2.2	2.1–2.8	2.5–3.4	3.1–4.0	4.0–5.1	15	20	ns	10	0–0
Children, both sexes, 7–10 years	311	1.8–2.4	2.3–3.1	2.7–3.7	3.3–4.3	4.2–5.5	15	20	ns	10	0–0
Males, 11–14 years	49	2.0–2.7	3.0–3.8	3.8–4.9	4.9–6.2	7.1–8.9	15	20	ns	10	0.6–2.5
Females, 11–14 years	51	1.2–1.7	1.8–2.6	2.5–3.5	3.5–4.6	5.6–7.0	15	20	ns	10	0.3–0.7
Males, 15–17 years	56	1.9–2.5	2.8–3.7	3.8–4.9	5.0–6.5	7.6–9.8	15	20	ns	10	1.1–4.5
Females, 15–17 years	58	1.0–1.2	1.7–2.1	2.5–3.1	3.6–4.7	6.1–8.3	15	20	ns	10	0.5–2.5
Males, 18–64 years	793	2.0–2.6	3.0–3.9	3.9–5.1	5.2–6.7	7.6–9.9	15	20	ns	10	1.0–4.8
Females, 18–64 years	873	1.4–1.9	2.1–2.7	2.8–3.5	3.7–4.6	5.6–6.7	15	20	ns	10	0.1–0.3
Males, ≥65 years	80	1.6–2.2	2.5–3.4	3.5–4.6	4.7–6.2	7.3–9.5	15	20	ns	10	1.0–3.9
Females, ≥65 years	123	2.1–2.6	2.7–3.3	3.1–3.9	3.6–4.5	4.4–5.7	15	20	ns	10	0–0

^(1)^ Lower bound (LB) and upper bound (UB) for the sum of vitamin D_2_ + D_3_. ^(2)^ An AI is set when scientific evidence is insufficient to set an EAR. ^(3)^ When the median intake (50th percentile) of the population is above the AI, the risk for inadequate intake is low. No statement (ns) is formulated on the adequacy of vitamin D when the median intake is below the AI. ^(4)^ Usual intake was for >95th percentile individuals of all sex/age categories lower than the EAR. * All requirements are set based on conditions of minimal sun exposure (i.e., only from diet). AI: Adequate Intake; EAR: Estimated Average Requirement; EFSA: European Food Safety Authority; DACH: German-speaking countries (Deutschland, Austria, Confoederatio Helvetica); IoM: Institute of Medicine.

**Table 3:** Main dietary sources of vitamin D in 10 Czech population groups.

Sex/Age Category	Food	% Contribution to Usual Dietary Intake of Vitamin D	Sex/Age Category	Food	% Contribution to Usual Dietary Intake of Vitamin D
**Children, 4–6 years**	Hen eggs	20.9	**Children, 7–10 years**	Hen eggs	21.4
	Fine bakery wares (excluding biscuits)	15.3		Fine bakery wares (excluding biscuits)	19.2
	Cow’s milk	12.5		Margarines	10.4
	Margarines	12.4		Cow’s milk	9.8
	Cocoa drinks powder	5.5		Butter	5.1
	∑	66.6		∑	65.9
**Males, 11–14 years**	Hen eggs	25.7	**Females, 11–14 years**	Hen eggs	22.7
	Margarines	15.8		Fine bakery wares (excluding biscuits)	17.5
	Fine bakery wares (excluding biscuits)	14.4		Margarines	13.1
	Cow’s milk	7.7		Cow’s milk	8.9
	Butter	5.1		Freshwater fish	5.8
	∑	68.7		∑	68.0
**Males, 15–17 years**	Hen eggs	23.8	**Females, 15–17 years**	Hen eggs	27.9
	Fine bakery wares (excluding biscuits)	14.6		Fine bakery wares excluding biscuits	12.4
	Margarines	8.6		Margarines	7.1
	Cow’s milk	5.4		Cow’s milk	6.4
	Marinated fish	5.3		Smoked fish	5.6
	∑	57.7		∑	59.4
**Males, 18–64 years**	Hen eggs	21.4	**Females, 18–64 years**	Hen eggs	23.3
	Fine bakery wares (excluding biscuits)	11.4		Fine bakery wares (excluding biscuits)	12.3
	Margarines	9.4		Margarines	12.3
	Salads *	6.2		Freshwater fish	8,0
	Pork meat	4.8		Salads *	4.2
	∑	53.2		∑	60.1
**Males, ≥65 years**	Hen eggs	22.6	**Females, ≥65 years**	Hen eggs	21.7
	Margarines	12.7		Margarines	17.6
	Fine bakery wares (excluding biscuits)	12.3		Fine bakery wares (excluding biscuits)	14.2
	Butter	5.3		Freshwater fish	9.8
	Marinated fish	5.2		Cow’s milk	5.4
	∑	58.1		∑	68.7

* Salads are composed of 50% of fish salad.

**Table 4:** Overview of vitamin D content in analyzed food groups.

Food Group	Number (*n*) of Kinds of Foods in a Group	Range of Vitamin D Content (Sum D_2_ + D_3_) μg/100 g Edible Portion
Fish and products	5	0.83–11.11
Milk-based infant formula powder	1	9.92
Cocoa drink powder	1	1.12
Chocolate and chocolate products	2	<LOQ *
Eggs	1	4.03
Milk and cream	4	0.16–0.53
Fermented milk products	5	<LOQ *–0.62
Dairy desserts	4	0.07–0.71
Cheese	7	0.05–0.85
Vegetable fats and oils	4	<LOQ *–8.17
Animal fats	2	<LOQ *–0.85
Meat and offal	10	<LOQ *–1.73
Meat products	16	<LOQ *–1.93
Cereals and flours	4	<LOQ *
Bread and similar products	5	<LOQ *
Fine bakery products	6	<LOQ *–1.09
Pasta and doughs	2	<LOQ *
Fruit, vegetables and cultivated mushrooms	5	<LOQ *
Miscellaneous foods	2	0.25–2.99

* LOQ: limit of quantification (LOQ D_2_—0.39 μg/kg; LOQ D_3_—0.18 μg/kg).
